# Longitudinal Serum Proteome Characterization of COVID-19 Patients With Different Severities Revealed Potential Therapeutic Strategies

**DOI:** 10.3389/fimmu.2022.893943

**Published:** 2022-07-26

**Authors:** Songfeng Wu, Yuan Xu, Jian Zhang, Xiaoju Ran, Xue Jia, Jing Wang, Longqin Sun, Huan Yang, Yulei Li, Bin Fu, Changwu Huang, Pu Liao, Wei Sun

**Affiliations:** ^1^ State Key Laboratory of Proteomics, Beijing Proteome Research Center, National Center for Protein Sciences (Beijing), Beijing Institute of Lifeomics, Beijing, China; ^2^ Department of Clinical Laboratory, Chongqing General Hospital, Chongqing, China; ^3^ Department of Clinical Laboratory, Affiliated Hospital of North Sichuan Medical College, Nanchong, China; ^4^ Chongqing Medical University, Chongqing, China; ^5^ Department of Clinical Laboratory, Chongqing Public Health Medical Center, Southwest University Public Health Hospital, Chongqing, China; ^6^ Beijing Qinglian Biotech Co., Ltd, Beijing, China; ^7^ School of Clinical Medicine, Southwest Medical University, Luzhou, China; ^8^ Savaid Medical School, University of Chinese Academy of Sciences, Beijing, China; ^9^ Department of Clinical Laboratory, Chongqing Fifth People’s Hospital, Chongqing, China

**Keywords:** COVID-19, proteomics, serum, severity, heterogeneity, progression

## Abstract

The COVID-19 pandemic caused by SARS-CoV-2 is exerting huge pressure on global healthcare. Understanding of the molecular pathophysiological alterations in COVID-19 patients with different severities during disease is important for effective treatment. In this study, we performed proteomic profiling of 181 serum samples collected at multiple time points from 79 COVID-19 patients with different severity levels (asymptomatic, mild, moderate, and severe/critical) and 27 serum samples from non-COVID-19 control individuals. Dysregulation of immune response and metabolic reprogramming was found in severe/critical COVID-19 patients compared with non-severe/critical patients, whereas asymptomatic patients presented an effective immune response compared with symptomatic COVID-19 patients. Interestingly, the moderate COVID-19 patients were mainly grouped into two distinct clusters using hierarchical cluster analysis, which demonstrates the molecular pathophysiological heterogeneity in COVID-19 patients. Analysis of protein-level alterations during disease progression revealed that proteins involved in complement activation, the coagulation cascade and cholesterol metabolism were restored at the convalescence stage, but the levels of some proteins, such as anti-angiogenesis protein PLGLB1, would not recovered. The higher serum level of PLGLB1 in COVID-19 patients than in control groups was further confirmed by parallel reaction monitoring (PRM). These findings expand our understanding of the pathogenesis and progression of COVID-19 and provide insight into the discovery of potential therapeutic targets and serum biomarkers worth further validation.

## Introduction

Coronavirus Disease-2019 (COVID-19) caused by severe acute respiratory syndrome coronavirus 2 (SARS-CoV-2) was declared a pandemic by the World Health Organization on 11 March 2020 (1). As of 1 June 2022, the cumulative number of confirmed cases of COVID-19 in the world has exceeded 500 million. The development of efficient therapies for treating COVID-19 remains critical for epidemic control.

To date, remdesivir, dexamethasone, and vaccines are considered treatment or prevention options for COVID-19 to shorten the recovery time, improve clinical status, and reduce the mortality rate (2–4). However, there are still many problems to be solved for treating COVID-19. First, new medications with more definitive and specific effects for severe COVID-19 patients are still needed. Besides, the pathogenic mechanism in asymptomatic patients, who account for 80% of SARS-CoV-2 infections (5), remains unclear. Understanding how the body responds to infection without obvious symptoms will enlighten the treatment for other symptomatic patients, particularly in their early infection stage. Additionally, many patients who have recovered from COVID-19 suffer from long-term sequelae, even in mild cases (6). The underlying molecular mechanism of the long-term effects of COVID-19 needs to be discovered to improve the existing treatment strategy.

To answer the questions above, we analyzed longitudinal serum samples from COVID-19 patients (including asymptomatic, mild, moderate, severe, and critical patients, collected at multiple time points) and control individuals (including healthy control and non-COVID-19 patients with fever/cough symptoms) using a tandem mass tag (TMT) stable isotope labeled proteomics strategy. Based on our proteome data, we deciphered the serum molecular characteristics of COVID-19 patients. For the first time, the heterogeneity of COVID-19 patients based on serum proteome and serum protein alterations related to disease progression at all severity levels was analyzed. The findings of this study might be helpful for comprehensively understanding the molecular mechanisms of COVID-19 and provide insight into potential therapeutic strategies for this disease.

## Results

### Samples and Proteomic Profiling

**Figure 1:**
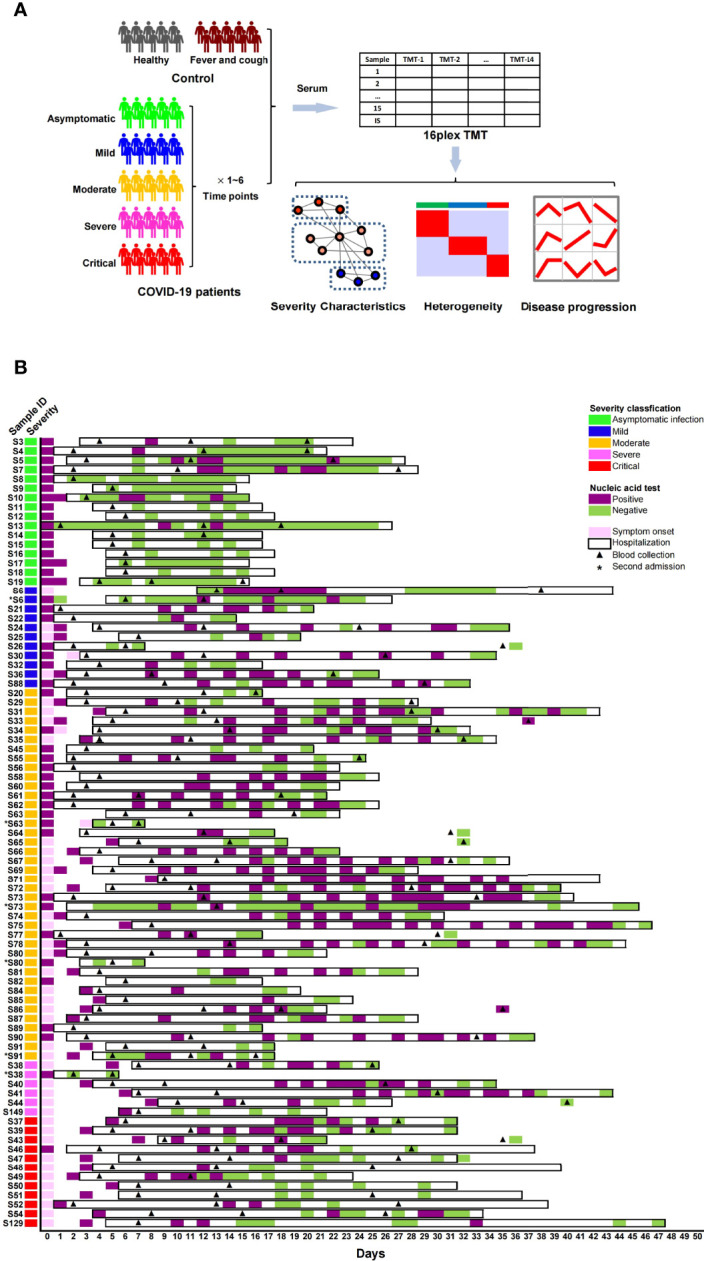
Proteome analysis of serum samples from COVID-19 patients and control groups. **(A)** Study design for sampling, proteomic quantification, and data analysis of serum samples from COVID-19 patients. **(B)** Basic information of sample collection from COVID-19 patients.

The serum samples were obtained from 79 diagnosed COVID-19 patients, including asymptomatic (n = 16), mild (n = 10), moderate (n = 36), severe (n = 5) and critical (n = 12) cases, 13 non-COVID-19 patients with fever/cough symptoms, and 14 healthy individuals (**Figure 1A**). Detailed clinical information of the participants is shown in **Table 1** and **Table S1**. From each symptomatic and asymptomatic COVID-19 patient, serum samples at an early stage (mainly within the first week after the onset of symptoms or the first nucleic acid positive test) were collected (**Figure 1B**). To identify longitudinal alterations of proteins, additional serum samples from most of the COVID-19 patients (n = 43) at progression and convalescence stages were also obtained (**Figure 1B**).

**Table 1:** Demographics of COVID-19 patients and the control group.

Variables			COVID-19					
	Healthy Control (n = 14)	Non-COVID-19 (n = 13)	Total (n = 79)	Asymptomatic(n = 16)	Mild (n = 10)	Moderate (n = 36)	Severe (n = 5)	Critical (n = 12)
Sex-n. (%)								
Male Female	9 (64.3) 5 (35.7)	7 (53.8) 6 (46.2)	38 (48.1) 41 (51.2)	5 (31.3) 11 (68.8)	6 (60.0) 4 (40.0)	17 (47.2) 19 (52.8)	1 (20.0) 4 (80.0)	9 (75.0) 3 (25.0)
Age-year								
M ± SD. Median (IQR) Range	42.9 ± 11.8 44.5 (33.3–50.0) 24.0–65.0	44.3 ± 16.1 39.0 (35.0–52.0) 22.0–78.0	43.8 ± 17.1 45.0(34.0–55.5) 4.0–80.0	38.7 ± 15.9 39.5 (27.5–48.5) 5.0–67.0	25.5 ± 18.5 19.5 (13.8–31.3) 4.0–58.0	43.6 ± 13.5 44.0 (35.0–50.0) 19.0–78.0	63.0 ± 12.3 65.0 (56.0–70.0) 44.0–80.0	56.5 ± 12.1 58.0 (48.8–66.5) 36.0–74.0
Days from Admission to Severe/Critical Symptoms								
M ± SD.			4.1 ± 4.0				2.0 ± 3.5	4.9 ± 3.9
Median (IQR)			2.0 (1.0–9.0)				0.0 (0.0–1.0)	4.5 (1.0–9.3)
Range			0.0–11.0				0.0–9.0	0.0–11.0
Days from Admission to Discharge ^a^								
M ± SD.			21.8 ± 9.5	15.5 ± 5.8	21.4 ± 8.8	22.8 ± 9.7	20.0 ± 10.4	28.6 ± 7.7
Median (IQR)			20.0 (13.0–29.0)	12.0 (12.0–20.0)	21.0 (13.5–31.0)	21.5 (15.8–28.3)	17.5 (15.5–27.0)	28.0 (25.0–35.0)
Range			4.0–43.0	10.0–27.0	6.0–32.0	4.0–43.0	4.0–36.0	12.0–42.0
Days from Onset/First Nucleic Acid Positive (Asymptomatic) to First Consecutive Nucleic Acid Negative ^a^								
M ± SD.			20.6 ± 10.5	10.4 ± 6.0	22.0 ± 9.8	23.5 ± 10.1	21.0 ± 12.2	22.8 ± 7.9
Median (IQR)			20.0 (14.0–27.0)	8.5 (6.8–14.0)	22.0 (15.0–31.5)	21.0 (15.8–30.5)	22.0 (12.8–29.8)	20.0 (18.0–25.5)
Range			2.0–44.0	2.0–23.0	5.0–37.0	4.0–44.0	2.0–38.0	14.0–44.0
Days from Onset/First Nucleic Acid Positive (Asymptomatic) to Discharge ^a^								
M ± SD.			25.4 ± 9.9	18.6 ± 4.8	24.7 ± 10.3	26.1 ± 10.0	29.8 ± 7.8	33.2 ± 6.7
Median (IQR)			24.0 (17.0–32.0)	16.5 (15.0–21.5)	25.0 (17.5–33.0)	23.5 (18.8–32.5)	26.0 (25.0–34.0)	32.0 (31.0–37.3)
Range			5.0–47.0	14.0–28.0	7.0–44.0	7.0–46.0	21.0–43.0	21.0–47.0
Symptoms-no. (%)								
Fever		13 (100.0)	47 (59.5)	0 (0.0)	5 (50.0)	26 (72.2)	4 (80.0)	12(100.0)
Cough		5 (38.5)	42 (53.2)	0 (0.0)	5 (50.0)	23 (63.9)	4 (80.0)	10 (83.3)
Expectoration		0 (0.0)	24 (30.4)	0 (0.0)	3 (30.0)	12 (33.3)	3 (60.0)	6 (50.0)
Pharyngalgia		4 (30.8)	12 (15.2)	0 (0.0)	3 (30.0)	8 (22.2)	0 (0.0)	1 (8.3)
Headache		0 (0.0)	16 (20.3)	0 (0.0)	3 (30.0)	5 (13.9)	1 (20.0)	7 (58.3)
Fatigue		3 (23.1)	22 (27.8)	0 (0.0)	2 (20.0)	7 (19.4)	4 (80.0)	9 (75.0)
Diarrhea		1 (7.7)	17 (21.5)	0 (0.0)	2 (20.0)	11 (30.1)	1 (20.0)	4 (33.3)
Chest Tightness		0 (0.0)	15 (19.0)	0 (0.0)	2 (20.0)	4 (11.1)	2 (40.0)	7 (58.3)
Rash		0 (0.0)	6 (7.6)	0 (0.0)	0 (0.0)	3 (8.3)	0 (0.0)	3 (25.0)
Basic Diseases-no. (%) ^b^								
Hypertension			9 (11.4)	3 (18.8)	2 (20.0)	1 (2.8)	0 (0.0)	3 (25.0)
Diabetes			5 (6.3)	0 (0.0)	0 (0.0)	0 (0.0)	3 (60.0)	2 (16.7)
Hematological System Diseases			4 (5.1)	1 (6.3)	1 (10.0)	0 (0.0)	1 (20.0)	1 (8.3)
Digestive System Diseases			7 (8.9)	1 (6.3)	0 (0.0)	4 (11.2)	0 (0.0)	2 (16.7)
Other Cardiovascular System Diseases			2 (2.5)	1 (6.3)	0 (0.0)	0 (0.0)	0 (0.0)	1 (8.3)
Respiratory System Diseases			1 (1.3)	0 (0.0)	0 (0.0)	0 (0.0)	1 (20.0)	0 (0.0)
Other Metabolic Diseases			3 (3.8)	1 (6.3)	0 (0.0)	1 (2.8)	0 (0.0)	1 (8.3)
Comorbidities-no. (%) ^c^								
Respiratory System			21 (26.6)	0 (0.0)	0 (0.0)	6 (16.7)	3 (60.0)	12 (100.0)
Digestive System			13 (16.5)	1 (10.0)	1 (10.0)	6 (16.7)	1 (20.0)	4 (33.3)
Metabolic Diseases			2 (2.5)	0 (0.0)	0 (0.0)	1 (2.8)	0 (0.0)	0 (0.0)
Hematological System			5 (6.3)	0 (0.0)	2 (20.0)	2 (5.6)	0 (0.0)	1 (8.3)
Immune System			4 (5.1)	0 (0.0)	0 (0.0)	1 (2.8)	1 (20.0)	2 (16.7)
Urinary System			1 (1.3)	0 (0.0)	0 (0.0)	0 (0.0)	0 (0.0)	1 (8.3)
Neuropsychological System			2 (2.5)	0 (0.0)	1 (10.0)	0 (0.0)	1 (20.0)	0 (0.0)
Skin Diseases			3 (3.8)	0 (0.0)	0 (0.0)	0 (0.0)	0 (0.0)	3 (25.0)
Cardiovascular System			5 (6.3)	0 (0.0)	0 (0.0)	2 (5.5)	1 (20.0)	2 (16.7)
Other Diseases			5 (6.3)	0 (0.0)	0 (0.0)	1 (2.8)	1 (20.0)	3 (25.0)
Treatment-no. (%)								
Oxygen Inhalation			45 (57.0)	0 (0.0)	5 (50.0)	23 (63.9)	5 (100.0)	12 (100.0)
Antibiotics			20 (25.3)	0 (0.0)	1 (10.0)	6 (16.7)	4 (80.0)	9 (75.0)
Antiviral Drug			77 (97.5)	14 (87.5)	10 (100.0)	36 (100.0)	5 (100.0)	12 (100.0)
Methylprednisolone			14 (17.7)	0 (0.0)	0 (0.0)	2 (5.5)	2 (40.0)	10 (83.3)
Chinese Medicine			62 (78.5)	14 (87.5)	10 (100.0)	23 (83.9)	3 (60.0)	12 (100.0)
Immunomodulator/Immunoglobulin			16 (20.3)	1 (6.3)	1 (10.0)	3 (8.3)	3 (60.0)	8 (66.7)
Plasma of Convalescent Patients			2 (2.5)	0 (0.0)	0 (0.0)	0 (0.0)	1 (20.0)	1 (8.3)
Chest CT-no. (%)								
Abnormality of Chest Radiographs			59 (74.7)	6 (37.5)	0 (0.0)	36 (100.0)	5 (100.0)	12 (100.0)

a no. (%), number; SD, standard deviation; IQR, interquartile range. For patients who hospitalized twice, the days were calculated twice as two separated hospitalizations.

b Basic diseases mean diseases occurred before SARS-CoV-2 infection, according to the admission diagnosis.

c Comorbidities mean diseases occurred after SARS-CoV-2 infection, according to the admission and discharge diagnosis and laboratory tests during the hospitalization.

All serum samples from 106 individuals underwent proteomic analysis, and 723 proteins were quantified by the TMT method (**Table S2**). The proteomes of the early-stage samples were used to identify protein characteristics related to the severity and molecular heterogeneity of COVID-19, while the proteomes of samples from three stages were analyzed to identify proteins with level alterations during disease progression (**Figure 1A**).

### Dysregulated Immune Response and Metabolism Reprogramming in Severe and Critical COVID-19 Patients

**Figure 2:**
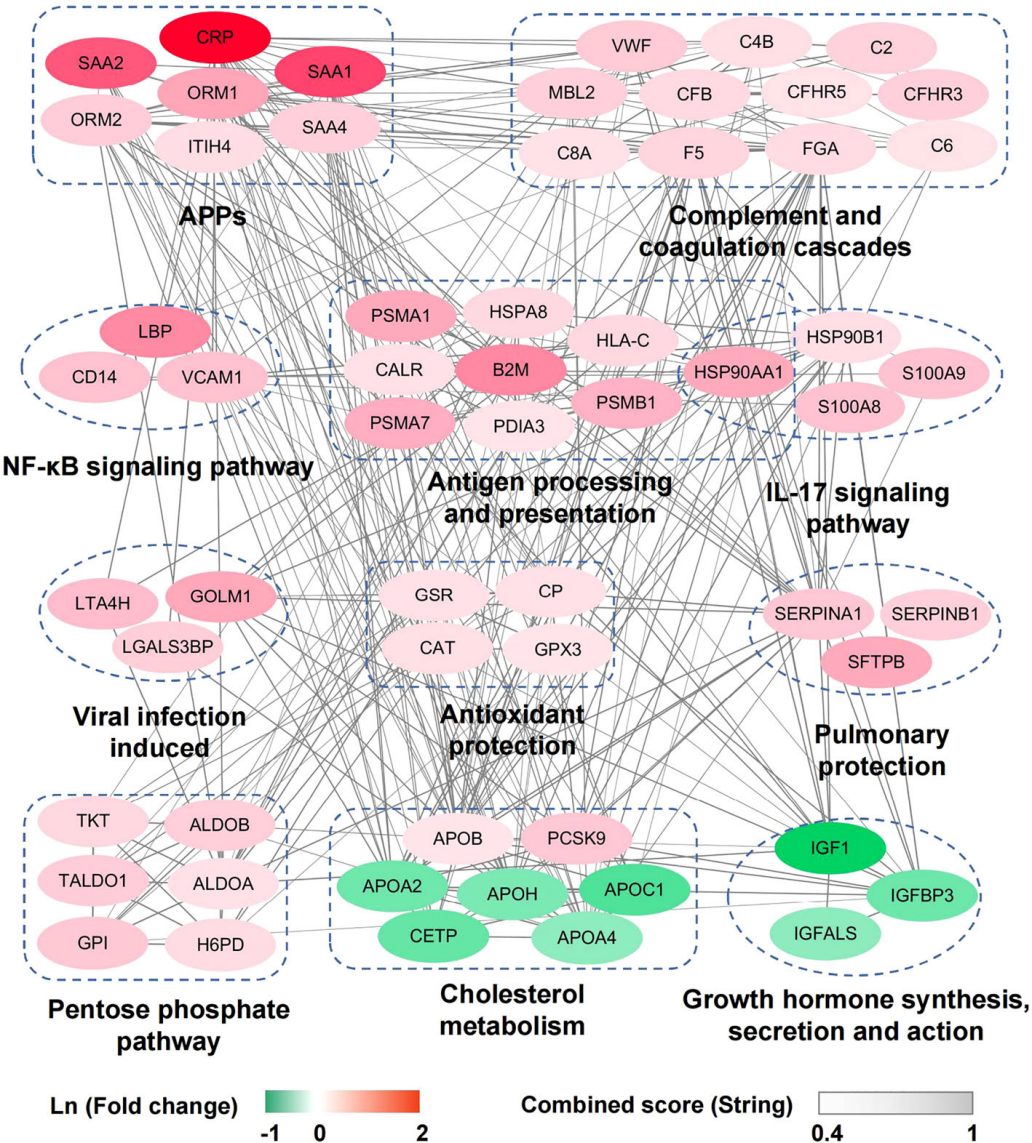
Network of dysregulated proteins in enriched pathways and important functional groups in severe and critical patients compared with other COVID-19 patients. The dysregulated proteins in severe and critical patients are mainly grouped into immune response and metabolism reprogramming pathways/functional groups and are connected in the protein-protein interaction network according to the String database. The color of each protein corresponds to Ln (fold change) between severe/critical and other patients, and the gray value of each line corresponds to the combined score between two connected proteins in String database.

To identify the molecular pathophysiological characteristics of severe/critical COVID-19 patients, the early-stage serum proteomes of severe and critical patients were compared with those of other COVID-19 patients and differential proteins (|fold change|>1.2, *P <*0.05) were used for further analysis (**Table S3**). Remarkable elevation of acute phase proteins (APPs) (CRP, SAA1, SAA2, SAA4, ORM1, ORM2, and ITIH4), and complement activation and coagulation proteins (MBL2, CFB, CFHR3, CFHR5, C2, C4B, C6, C8A, F5, VWF, and FGA) were detected in severe and critical patients (**Figure 2**). Along with these proteins, several other viral infection induced proteins such as LGALS3BP, GOLM1, and LTA4H were also increased (**Figure 2**). Meanwhile, we found marked elevation of antioxidant protection proteins (GPX3, GSR, CAT, and ceruloplasmin) and pulmonary protection proteins such as proteolytic destruction inhibitors (SERPINA1 and SERPINB1) and alveolar stabilizer (SFTPB) in severe and critical COVID-19 patients (**Figure 2**), suggesting the induction of a body self-protection mechanism. These results reflected exuberant immune responses and hyperactivity of the complement and coagulation systems at the early stage in severe and critical COVID-19 patients.

We then performed pathway enrichment analysis based on KEGG (Kyoto Encyclopedia of Genes and Genomes) annotation. Inflammatory response pathways (NF-κB signaling pathway, IL-17 signaling pathway, and antigen processing and presentation pathway) and pentose phosphate pathway were upregulated in severe and critical COVID-19 patients (**Figure 2**). This finding presents the hyperactivity of the inflammatory response state and dysregulation of metabolism in severe and critical COVID-19 patients. The upregulated proteins in the antigen processing and presentation pathway (HSP90, HLA-C, HSPA8, CALR, PDIA3, and B2M) are mainly involved in the MHC-I antigen processing system. Considering that MHC-I with viral antigen activates CD8^+^ T cells and natural killer (NK) cells, we speculate that the hyperactivity of the MHC-I antigen processing system may lead to exhaustion of CD8^+^ T cells. Consistent with this, the decrease in CD8^+^ T cells in severe and critical patients was detected in our data (**Table S4**), which was supported by other studies (7, 8). In addition to those upregulated pathways, cholesterol metabolism reprogramming was found in our data (**Figure 2**). Although the cholesterol metabolism pathway was significantly downregulated (including APOA2, APOC1, APOH, CETP, and APOA4), the increased levels of PCSK9 and APOB suggested the dysregulation of cholesterol metabolism in severe and critical COVID-19 patients. Intriguingly, marked downregulation of growth hormone synthesis, secretion, and action pathway in severe and critical COVID-19 patients was first identified (**Figure 2**).

### Effective Immune Response in Asymptomatic Patients

**Figure 3:**
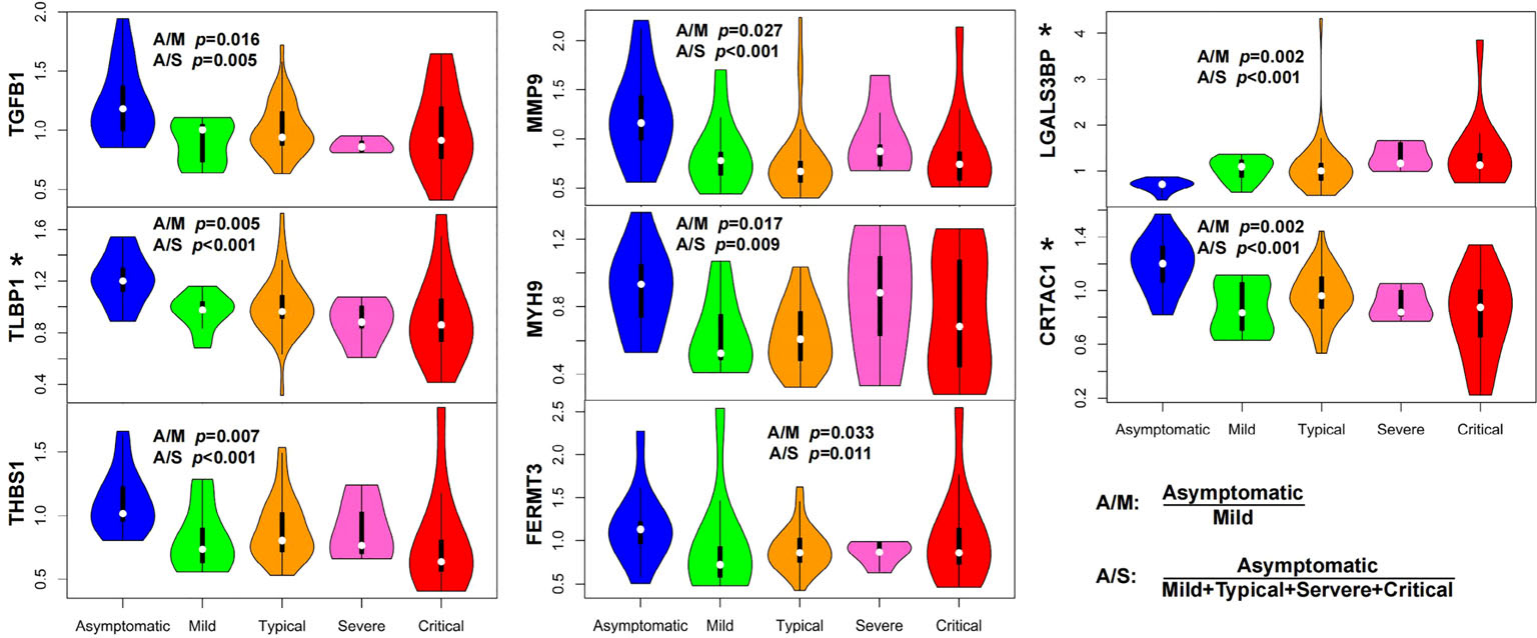
Characteristics of asymptomatic patients presented an immune balance in the body. The serum levels of proteins in TGF-β pathway (TGFB1, LTBP1, and THBS1) and proteins related to cytotoxic immune response (LGALS3BP) and leukocyte migration (MMP9, MYH9, and FERMT3) significantly changed (*P <*0.05, Wilcox nonparametric test) in asymptomatic patients compared with those in mild (A/M) or symptomatic COVID-19 patients (A/S). Elevation of LTBP1 and CRTAC1, and decline of LGALS3BP in asymptomatic patients were commonly found when compared with those in each symptomatic group (mild, moderate, severe, and critical), respectively (indicated with asterisks, *P <*0.05, Wilcox nonparametric test). (A/M means asymptomatic vs. mild; A/S means asymptomatic vs. symptomatic, the latter of which includes mild, moderate, severe, and critical patients.).

In contrast with severe and critical patients, asymptomatic patients represent the best outcome without clinical symptoms and further deterioration of the disease process. Understanding how the body responds to infection in asymptomatic patients is also very inspiring for treating this disease, particularly in its early stages. We first compared the early-stage serum proteomes of asymptomatic patients with those of mild patients. We found that the levels of proteins involved in TGF-β pathway (TGFB1, LTBP1, and THBS1) and proteins contributing to leukocyte migration (MMP9, MYH9, and FERMT3) increased in asymptomatic patients (**Figure 3**). The same result was also found compared with all symptomatic patients (**Figure 3**).

As a component of the large latent complex binding TGF-β to the extracellular matrix, LTBP1 is the key regulator of TGF-β (TGFB1, TGFB2, and TGFB3) that controls TGF-β secretion and activation (9). *In vivo*, THBS1, which releases active TGF-β from its latent form, is a major activator of TGF-β (10). The elevation of TGFB1, LTBP1, and THBS1 in asymptomatic patients indicated the potential role of TGF-β pathway in symptom control. Mechanistically, TGF-β regulates T-cell homeostasis by inhibiting the activation of self-reactive T cells (11), inducing T-regulatory (Treg) cells to differentiate from peripheral CD4^+^CD25^−^ naive T cells and mediating immunosuppressive function of Treg cells (12, 13). That is, the downregulation of TGF-β or reduction of Treg cells may lead to pathological inflammation caused by aberrant T-cell response, which conforms to the fact that decreased responses of Treg cells in COVID-19 intensive care unit (ICU) patients is strongly related to hyperinflammation and disease pathogenesis (14). The elevation of proteins related to leukocyte migration (MMP9) (15), leukocyte cell motility (MYH9) (16, 17), and leukocyte adhesion to endothelial cells (FERMT3) (18) might contribute to leukocyte migration into infected tissues from the blood, which is crucial for defense against invading microbial pathogens (19). These results revealed an effective immune response in asymptomatic patients characterized by pathogen defense in immune homeostasis.

We also compared the serum proteomes of asymptomatic patients with those in each symptomatic group (mild, moderate, severe, and critical), respectively. Alterations of three proteins (elevation of LTBP1 and CRTAC1, and decline of LGALS3BP) in asymptomatic patients were commonly found in all comparisons (**Figure 3**, indicated with asterisks). The relationship between CRTAC1 and virus infection has not been reported. One study discovered that CRTAC1 was prominently expressed in alveolar type-2 epithelial (AT2) cells in the lungs, and it was a biomarker of AT2 cell health status in bronchoalveolar lavage fluid and plasma (20). The higher serum level of CRTAC1 in the asymptomatic group than in each of the symptomatic groups was consistent with the relatively healthy status of lung function in asymptomatic patients. LGALS3BP is a virus-induced protein, which could lead to the production of interferons and pro-inflammatory cytokines, including IL-6 and TNF-α (21). The elevation of IL-6 and TNF-α was shown to be associated with the severity of COVID-19 due to the cytokine storms (22), and remarkable elevation of plasma LGALS3BP was also observed in COVID-19 ICU patients (23). As such, the levels of CRTAC1 and LGALS3BP in asymptomatic patients might reflect the limited immune damage compared with symptomatic patients.

### Molecular Heterogeneity of COVID-19 Patients

**Figure 4:**
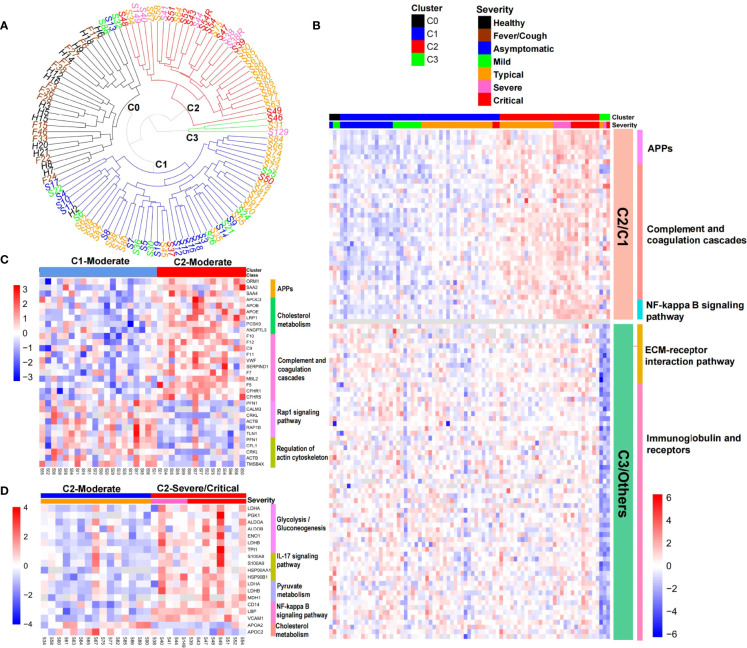
Heterogeneity of COVID-19 patients. **(A)** Hierarchical cluster analysis separated all the samples collected at the first time-spot into four clusters. **(B)** Heat map of representative protein and pathway alterations between COVID-19 patients in clusters C2 and C1, or C3 and other clusters. **(C)** Heat map of representative protein and pathway alterations between moderate patients in clusters C2 and C1. **(D)** Heat map of inflammation and metabolism pathway alterations between severe/critical and moderate patients in cluster C2.

COVID-19 is a heterogeneous disease, and a better understanding of patient heterogeneity could help clinicians supply precision treatment for COVID-19 patients (24). To explore the molecular pathophysiological heterogeneity in COVID-19 patients, we performed hierarchical cluster analysis based on the serum proteome data from the early stage of COVID-19 patients and control groups. All the individuals were separated into four clusters (C0, C1, C2, and C3) (**Figure 4A**). Healthy controls (except one sample being in C1) and non-COVID-19 patients with fever/cough were clustered into C0. COVID-19 patients were distributed into four clusters. The majority of asymptomatic and mild COVID-19 patients were in C1, with three patients being in C0. All severe COVID-19 patients and the most critical patients were distributed in C2, again with four critical patients being in C1 or C3. Surprisingly, moderate COVID-19 patients were almost equally distributed in C1 and C2, with only one patient being in C3. These results demonstrate the molecular heterogeneity in COVID-19 patients, particularly in moderate patients.

We then explored the molecular characteristics of these clusters. By comparing with C1, we found that C2 was characterized by the elevation of APPs, complement and coagulation cascades, NF-kB signaling pathway (**Figure 4B**), and a declining trend of growth hormone synthesis, secretion, and action pathway (*p* = 0.013, not shown in **Figure 4B**), which was consistent with the characteristics of severe and critical patients (**Figure 2**).

Because moderate patients were mainly divided into C1 (n = 20) and C2 (n = 15) (**Figure 4A**), we next explored the molecular differences between them. APPs and proteins involved in complement and coagulation cascades were still elevated in the moderate patients in C2 compared with those in C1 (**Figure 4C**), which was consistent with **Figure 4B**. However, we also found marked elevation of several other proteins associated with cholesterol metabolism (APOC3, APOB, APOE, LRP1, PCSK9, and ANGPTL3) and reduced levels of proteins involved in rap1 signaling pathway and regulation of the actin cytoskeleton in moderate patients in C2 (**Figure 4C**). It has been shown that Rap1 tightens the endothelial barrier dependent on adherence junctions and regulates the actin cytoskeleton to control endothelial barrier function (25). Depletion of Rap1 leads to the depression of basal barrier function and hyperpermeability such as vascular leakage (25). The downregulation of the Rap1 signaling pathway in moderate patients in cluster C2 might reflect the epithelial barrier breakdown during virus infection.

Although parts of the moderate patients were divided into C2 together with the majority of severe/critical patients, they did not develop into a severe stage. We then examined the difference between the moderate and severe/critical patients in C2. Compared with moderate patients, severe/critical patients exhibited significant upregulation of inflammation (IL-17 signaling pathway, NF-κB signaling pathway) and energy/substance metabolism (glycolysis/gluconeogenesis, pyruvate metabolism) pathways, and downregulation of cholesterol metabolism (APOA2 and APOC2) (**Figure 4D**), which was partly consistent with the characteristics of the severe and critical patients shown in **Figure 2**.

The outlier cluster C3 had only one moderate and two critical patients. Compared with other clusters, the levels of proteins involved in the ECM-receptor interaction pathway, as well as immunoglobulin and receptors, markedly declined in C3 (**Figure 4B**). Integrin α2β1 induces platelet aggregation by mediating adhesion of platelets to different collagens (26). Declines of integrin ITGB1, proteins promoting the formation of platelet plugs (GP1BA, THBS1, GP5, and DAG1), and extracellular matrix proteins (COL1A1, COL6A1, COL6A3, and TNC) (**Table S3**) indicated decreased platelet aggregation in cluster C3. This conformed to the coagulation disorder or thrombocytopenia diagnosed in two of the three patients in cluster C3. Surprisingly, a quarter of the declined proteins in C3 were immunoglobulins (**Figure 4B**). This result might indicate the impaired humoral immunity and consequent immune effect in C3 patients. Consistent with this, the levels of immunoglobulin receptors FCGR2A and FCGR3A also significantly declined in C3 (**Table S3**).

### Serum Protein Alterations Related to the Disease Progression

**Figure 5:**
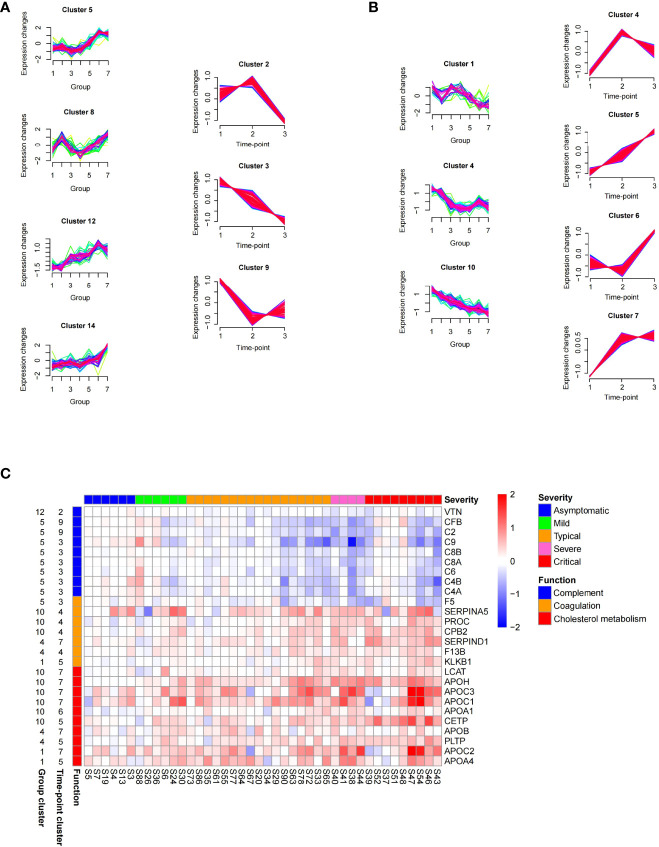
Time series proteomics data analysis of serum samples from COVID-19 patients. **(A)** Proteins whose level elevated with the increasing of severity (in group clusters 5, 8, 12, and 14 on the left) and declined at the late course of disease (in time-point clusters 2, 3, and 9 on the right). The numbers 1–7 in X-axis of group clusters stands for 7 sample groups (healthy, fever, asymptomatic, mild, moderate, severe, and critical). The numbers 1–3 in X-axis of time-point clusters represents 3 sampling time points. **(B)** Proteins whose level declined with the increasing of severity (in group clusters 1, 4, and 10 on the left) and elevated at the late course of disease (in time-point clusters 4, 5, 6, and 7 on the right). **(C)** Abundance changes of proteins in the enriched pathways between time-point 3 and time-point 1 (log2 fold change of time-point 3/time-point 1), which were shown for individual patients stratified by severity classification of COVID-19. The panel on the left of the heat map provides functional annotation for the proteins. The number on the left of the heat map represents the number of group cluster and time-point cluster of each protein.

To observe the alterations of serum proteins in COVID-19 patients, we examined protein levels related to the severity of COVID-19 and performed time-series analysis. The proteins that were identified in the serum samples from the early stage (the first time-point) of COVID-19 and controls were clustered into 16 group clusters using mFuzz (**Table S5**). Levels of proteins in group clusters 5, 8, 12, and 14 elevated significantly with the severity of COVID-19 (**Figure 5A**, left panel), while proteins in group clusters 1, 4, and 10 showed an opposite trend (**Figure 5B**, left panel). Meanwhile, we examined the longitudinal alterations of proteins related to the course of COVID-19. We found that proteins identified in the serum samples obtained from all three stages were separated into 9 time-point clusters (**Table S5**). Proteins in time-point clusters 2, 3, and 9 were decreased at the convalescence stage (**Figure 5A**, right panel), whereas proteins in clusters 4, 5, 6, and 7 increased (**Figure 5B**, right panel).

We are interested in the proteins that were increased or decreased with the severity of COVID-19 at the early stage but restored at the convalescence stage (common proteins in both groups and time-point clusters shown in **Figure 5A** or **Figure 5B**). Among these proteins, 80 proteins significantly changed (FDR <0.05) between time-point 3/time-point 1 (**Table S5**) were identified as signature proteins that varied with COVID-19 severity and alleviated during convalescence. Pathway enrichment analysis showed that complement and coagulation cascades and cholesterol metabolism were significantly enriched in those signature proteins (**Figure 5C**). Proteins related to complement activation (C2, C4A, C4B, C6, C8A, C8B, C9, CFB, and VTN) increased with the severity of COVID-19 but decreased over time. Conversely, components of the coagulation cascade (SERPINA5, SERPIND1, PROC, CPB2, F13B, and KLKB1) and proteins involved in cholesterol metabolism were downregulated with the severity of COVID-19 but increased over time. The changes of these proteins will be helpful for understanding the pathology of COVID-19 and have the potential value for monitoring disease progression or treatment responses.

Of note, some proteins could not be recovered in the majority of patients during the disease progression (**Table S6**). Among them, plasminogen-like protein B (PLGLB1, also known as PRP-B) exhibited the most pronounced trend that kept a high level in COVID-19 patients, and its level was even increased at the convalescence stage (time-point 3) compared with the early stage (time-point 1) (**Table S6**). PLGLB1 has an anti-angiogenesis function through inhibition of endothelial cell proliferation and blood tube formation (27), which may impede the rehabilitation of blood vessels damaged by SARS-CoV-2 infection. The possible relationship between these unrestored proteins and the long-term effects on the human body after SARS-CoV-2 infection deserves further study.

### Candidate SARS-CoV-2 Infection Biomarker With High Stability During the Course of Disease

**Figure 6:**
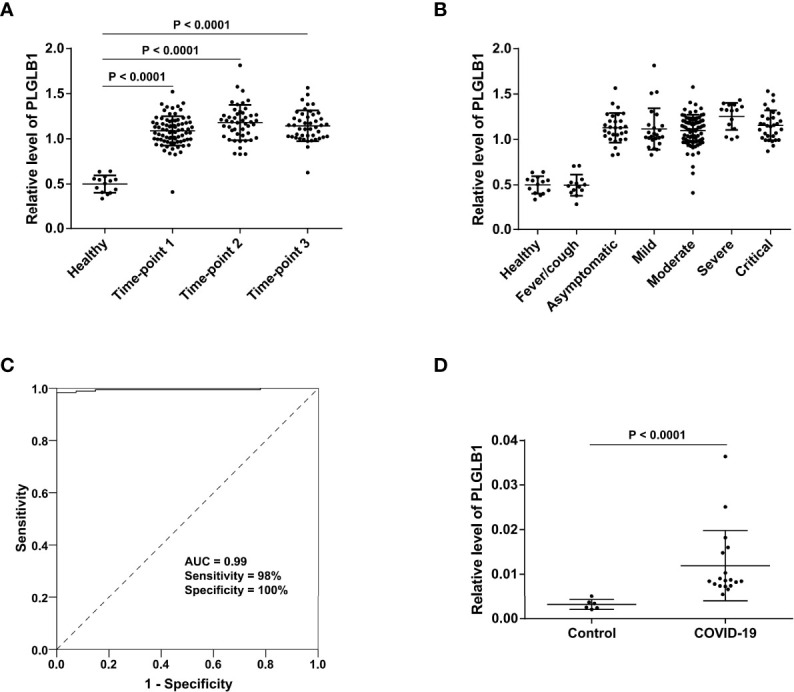
Serum level of PLGLB1 in COVID-19 patients and control individuals. **(A)** PLGLB1 maintained higher level during the three stages of COVID-19 (corresponding to time-points 1 to 3) compared with healthy controls. (*P <*0.0001, Wilcox nonparametric test). **(B)** PLGLB1 was significantly upregulated in all the COVID-19 groups (including asymptomatic group) compared with both non-COVID-19 controls (*P <*0.0001, Wilcox nonparametric test). **(C)** ROC curves for PLGLB1 to discriminate COVID-19 patients from non-COVID-19 controls. The result showed that the serum level of PLGLB1 could differentiate COVID-19 patients from uninfected individuals with an AUC of 0.99, a sensitivity of 98% and a specificity of 100%. **(D)** The PRM result validated that the serum level of PLGLB1 was significantly higher in COVID-19 patients than that in controls (*P <*0.0001, Wilcox nonparametric test).

It was interesting that PLGLB1 not only maintained a higher level during the three stages of COVID-19 compared with healthy controls (**Figure 6A**), but also increased in all severity groups (including the asymptomatic group) compared with non-COVID-19 controls (**Figure 6B**). The receiver operating characteristic curve (ROC) analysis showed that the serum level of PLGLB1 could stably differentiate uninfected individuals from COVID-19 patients (area under curve (AUC) = 0.99, sensitivity = 98%, specificity = 100%), no matter from which disease stage or severity the sample was collected (**Figure 6C**). Additionally, for 28 serum samples collected before consecutive negative nucleic acid tests, SARS-CoV-2 virus nucleic acid was also detected on the same day of serum sampling. Among those serum samples, the sensitivity of PLGLB1 was 96% (27/28), while the positive rate of simultaneous nucleic acid detection was only 68% (19/28).

Parallel reaction monitoring (PRM) validation of PLGLB1 was further performed in 24 serum samples (18 COVID-19 samples and 6 controls, including 17 new samples not included in the TMT experiment). The results showed that the serum level of PLGLB1 was significantly higher in COVID-19 patients than that in the controls (**Figure 6D**). These data suggest that PLGLB1 could be a candidate SARS-CoV-2 infection biomarker with high stability during disease.

## Discussion

Since there is a high mortality rate in critically ill COVID-19 cases (28), most of the previous proteomics studies on serum or plasma were focused on the severe patients, compared with mild or non-severe patients (29–31). In this study, we analyzed serum proteomes of COVID-19 patients with different severity and at different disease stages. Our data offer a proteomic landscape of serum changes induced by SARS-CoV-2 infection and comprehensively decipher the molecular pathophysiological characteristics and heterogeneity of COVID-19 patients.

In line with other reports (22, 32), we found the elevation of complement and coagulation proteins and inflammatory signaling pathways in severe and critical COVID-19 patients. Dysregulation of metabolism was another characteristic (**Figure 2**). The elevation of the pentose phosphate pathway, which facilitates SARS-CoV-2 replication, shows that targeting these metabolic pathways can help define novel treatment strategies for COVID-19 (33). It was interesting that though a majority of proteins in cholesterol metabolism decreased (APOA2, APOC1, APOH, CETP, and APOA4), PCSK9 and APOB were elevated in severe patients. Lipoproteins play a key role in viral infections beyond transporting hydrophobic lipids, and the levels of lipoprotein are modified during viral infections (34). APOA2, APOC1, and APOA4 are major apolipoproteins of HDL (high-density lipoprotein) particles. It was reported that HDLs might display antiviral effects by preventing virus penetration (35). Decreased levels of serum lipoproteins APOA2, APOC1, and APOA4, which associated with HDLs, may increase the pathogenicity of SARS-CoV-2 and prolong the virus clearance time (36). APOB, as a component in LDL (low-density lipoprotein) particles, was involved in viral infectivity (37). PCSK9, as a negative regulator of LDLR (LDL receptor), was also found to be increased in the PBMCs of COVID-19 patients and in the plasma of dengue virus infected patients with high viremia (38, 39). In hypoxic microenvironments, upregulation of PCSK9 induced by dengue virus reduces LDLR-mediated uptake of LDL cholesterol and augments virus infection by promoting *de novo* cholesterol synthesis in the endoplasmic reticulum, which inhibits STING-mediated antiviral response (39). Considering its inhibition of STING-mediated antiviral response, PCSK9 might be a potential target for the treatment of COVID-19 (40), in addition to being a known therapeutic target for hypercholesterolemia (41).

It is worth noticing that three growth hormone-related proteins (IGF1, IGFBP3, and IGFALS) were found to decline in severe and critical patients. A recent study (42) reported the decrease of serum IGF1 in ICU patients compared to mild patients. Reduced circulating levels of IGF1 or IGFALS were found to be associated with COVID-19 mortality (43) or in severe COVID-19 patients with adverse prognosis (44) in two separate studies. Although IGF1, IGFBP3, and IGFALS may be associated with diabetes (45–47), the levels of these proteins in our study in severe patients without diabetes were also very low, indicating that their decline was not only due to diabetes. Furthermore, these proteins all had the trend to decline with the severity and to increase in the late course of disease (belonging to group cluster 1 or 10, and time-point cluster 5 or 7) (**Table S5**). Epidemiological data from the pandemic has suggested that growth hormone deficiency might be associated with the severity of COVID-19 (48), but the molecular mechanisms remain unclear. The lower circulating levels of IGF1 and IGFBP3 were also associated with the incidence and mortality of adult respiratory distress syndrome (ARDS) (49). IGF1 also had immune-regulatory effects, including promoting the maturation of myeloid cells, priming phagocytes for the production of superoxide anions and cytokines, and it was found that in a mouse model, pretreatment with IGF1 improved survival in severe sepsis, and IGF1 treatment after infection rescued mice from severe sepsis (50). Considering the immune-regulatory function of IGF1 and its association with ARDS, our results suggest the therapeutic potential of IGF1 and that other endocrine changes in severe patients might also need to be given attention.

Asymptomatic infection represents the best outcome of COVID-19. For the first time, we found an effective immune response in asymptomatic patients characterized by pathogen defense in immune homeostasis with limited immune damage, which suggests the value of effective SARS-CoV-2 clearance in the immune balance to attenuate symptoms in infected patients. For example, maintaining the appropriate TGF-β pathway activity, leukocyte migration ability, and LGALS3BP level might be helpful for eliminating clinical symptoms at the early stage of infection.

Based on serum proteome profiling, we found that there were four clusters in COVID-19 patients. The cluster distribution of majority of patients was consistent with the clinical severity classification. However, the moderate patients were mainly divided into two clusters, which led to a more detailed understanding of the molecular characteristics and corresponding treatment of patients in this group.

It was unexpected that there was an outlier cluster C3 characterized by impaired humoral immunity and decreased platelet aggregation. Since hypoimmunity was not the basic disease of C3 patients, this change was more likely due to SARS-CoV-2 infection. Though the reason was unclear, the treatment of these patients might be different. For example, the high risk of co-infection or bleeding in these patients should be taken care of. Our study, for the first time, comprehensively deciphered the heterogeneity of COVID-19 patients based on serum proteome profiling and provided new molecular information for precision treatment.

The level alterations of serum proteins in complement and coagulation cascades and cholesterol metabolism were found to be related to COVID-19 severity and were restored during the disease course. It is interesting that under these circumstances, proteins in complement activation or coagulation cascades display opposite trends. The changes in these proteins may be used for monitoring disease. The unrestored proteins in the convalescence stage, such as PLGLB1, reminded us to pay attention to the possible long-term effects of SARS-CoV-2 infection.

One problem for the screening of SARS-CoV-2 infected patients is the instability of nucleic acid detection, which is a false negative due to the low viral load at the time of detection, or improper sample collection or preservation. Multiple and continuous nucleic acid tests are usually required for screening in high-risk populations. In this study, we found that the serum level of PLGLB1 could stably differentiate COVID-19 patients (ranging from one day to 4 months after diagnosis) from uninfected individuals with high accuracy. In contrast, the nucleic acid test results of COVID-19 patients fluctuated during the disease (as shown in **Table 1**). Both TMT and PRM results in this study suggested that serum levels of PLGLB1 could be a candidate SARS-CoV-2 infection biomarker with high stability. However, validation in an independent cohort is lacking in this study. In order to determine the reliability of this marker, further validation in multicenter cohorts is needed to exclude potential bias caused by enrollment, sample collection, and so on. Additionally, the healthy (n = 14) and the fever/cough (n = 13) control groups were relatively small in this study, and more samples in the control groups should be included to eliminate the possibility of overestimated performance of the candidate marker. Because this protein is not a direct readout of the virus (viral proteins, genome, etc.), but a host response to the infection, more control groups such as metabolic diseases, cancer, or other infections need to be included to evaluate the specificity of this candidate marker.

In summary, our data offered a proteome landscape of serum changes induced by SARS-CoV-2 infection and comprehensively deciphered the characteristics and heterogeneity of COVID-19 patients. These findings provided new inspiration at the molecular level for treatment and detection of COVID-19 patients in the future.

## Materials and Methods

### Patients and Sample Collection

Serum samples from 79 COVID-19 patients were collected from the Chongqing Public Health Medical Center, including patients with asymptomatic infection (n = 16) or with mild (n = 10), moderate (n = 36), severe (n = 5), and critical (n = 12) severity classification. All the patients were diagnosed with COVID-19 according to the Chinese Government Guideline (51). The SARS-CoV-2 virus nucleic acid detection (by real-time fluorescence RT-PCR) in nasopharyngeal swabs was all positive. The symptoms (such as fever, cough, fatigue, and chest tightness), severity classification, and other information are shown in **Tables 1**, **S1**. Classification of COVID-19 patients was performed according to the following guidelines: 1) Asymptomatic infected: SARS-CoV-2 virus detection or specific antibody positive but without clinical symptoms; 2) Mild: mild symptoms without pneumonia; 3) Moderate: fever or respiratory tract symptoms with pneumonia; 4) Severe (fulfill any of the three criteria): respiratory distress, respiratory rate ≥30 times/min; oxygen saturation ≤93% in resting state; arterial blood oxygen partial pressure (PaO_2_)/oxygen concentration (FiO_2_) ≤300 mmHg; and 5) Critical (fulfill any of the three criteria): respiratory failure and require mechanical ventilation; shock incidence; admission to ICU with other organ failure.

The serum samples were collected mainly at three time points during hospitalization or after discharge according to the Chinese Government Guideline (52): within the first week (point-1 at the early stage), within the second week (point-2 at the progression stage), and later than two weeks (point-3 at the convalescence stage) after the onset of symptoms or the first nucleic acid-positive test (for asymptomatic infected patients). For more than half of the COVID-19 patients (n = 43), serum samples at the three time points above were collected. For 8 patients with re-positive SARS-CoV-2 nucleic acid test results after discharge, extra serum samples at 1–3 time points after the first discharge were also collected. The sampling details of each patient are shown in **Figure 1B**.

Non-COVID-19 serum samples were collected as controls, including 13 samples from non-COVID-19 patients with similar clinical symptoms (fever and cough, collected from the Chongqing Fifth People’s Hospital, excluding SARS-CoV-2 infection by negative epidemiological history, laboratory tests, imaging examination, nucleic acid tests, IgM and IgG tests) and 14 samples from healthy individuals (collected from the Chongqing General Hospital, with no symptoms, negative nucleic acid tests for SARS-CoV-2 and blood routine tests).

For PRM validation, extra samples from 3 control individuals and 14 time-points from COVID-19 patients were also included.

The serum samples in this study are from a clinical trial registered in the Chinese Clinical Trial Registry (ChiCTR2000033872). This study was approved by the Ethical Review Board of Chongqing General Hospital.

### Sample Preparation and TMT Labeling

After 56 °C sterilization for 30 min (29), 5 μl of individual or pooled serum sample was denatured in 50 μl buffer containing 8 M urea in 50 mM Tris-Cl (pH 7.4) at 32 °C for 30 min. Then the sample was reduced with 10 mM dithiothreitol at 37 °C for 1 h, followed by alkylation for 30 min with 40 mM iodoacetamide in darkness at room temperature. After 4 times dilutions with 50 mM NH_4_HCO_3_, trypsin (Promega, 1/50 of protein amount) was added to the sample twice for digestion at 37 °C for 12 h and additional 4 h, respectively. The peptides were acidified with 0.1% trifluoroacetic acid, desalted with a homemade C18 (Agela Technologies, Tianjin, China) column, and dried by speed-vac. For each sample, the peptides were resolved with 200 μl of 200 mM triethylammonium bicarbonate (TEAB), and 25 μg of peptide was labeled with TMTpro 16plex label reagents (Thermo Fisher Scientific, San Jose, USA) according to the recommendations of the manufacturer. The sequentially collected samples from the same patient were arranged into the same TMT, and samples from different groups were included in each TMT (as shown in **Table S2**, with the pooled sample as the internal standard). The samples from each TMT were combined and dried by speed-vac.

### Liquid Chromatography Separation and Mass Spectrometry Data Acquisition

The TMT labeled peptides were separated by a L-3000 HPLC system (RIGOL, Beijing, China) with an XBridge Peptide BEH C18 column (5 μm, 25 cm × 4.6 mm) (Waters, Milford, MA, USA) using a gradient from 5 to 35% acetonitrile in water (pH adjusted to 10 with ammonia) at a flow rate of 1 ml/min. The separated peptides were combined into 40 fractions and dried by speed-vac. After being redissolved in 2% acetonitrile/0.1% formic acid (FA), the peptides were analyzed by a Q Exactive HF hybrid Quadrupole-Orbitrap (Thermo Fisher Scientific, San Jose, USA) coupled with a nano-HPLC instrument (UltiMate 3000 LC system, Dionex, CA, USA) in data-dependent acquisition (DDA) mode.

The peptides were loaded onto a precolumn and then analyzed using a 42 min LC gradient (from 4% to 40% buffer B) at a flow rate of 700 nl/min (analytical column, 1.9 μm, 120 Å, 150 mm × 150 μm i.d.). Buffer A was 0.1% FA in water, and buffer B was 80% acetonitrile and 0.1% FA in water. Full scan MS spectra (m/z 300–1,400, resolution 120,000) were acquired, followed by MS/MS (resolution 60,000) on the top 15 intense ions detected.

### Proteomics Data Processing

The mass spectrometry raw data were searched against a protein database composed of the *Homo sapiens* fasta (UniProtKB, 8 August 2020 downloaded, 20289 reviewed protein sequences) and the SARS-CoV-2 fasta (NCBI, version NC_045512.2) using Proteome Discoverer software (version 2.4.1.15, Thermo Fisher Scientific). The following parameters were applied: the precursor mass tolerance was 20 ppm; the fragment mass tolerance was 0.02 Da; the static modification was carbamidomethylation of cysteine; the variable modifications were TMTpro of lysine residues and N-termini of peptides, oxidation of methionine, and acetylation of N-termini of peptides; a maximum of two missed cleavages were allowed. The protein target false discovery rate (FDR) was 1%. Normalization was performed against the total peptide amount. The protein intensity of each sample in the same TMT batch was adjusted against the internal standard. Missing values were imputed with the minimum value only when proteins were quantified in no less than two-thirds of the samples in the same TMT batch.

### Quality Control of Proteome Data

To evaluate the quality of the proteome data, two samples were analyzed with intra-TMT (S54-1) and inter-TMT (S38-1) replications. A Spearman correlation analysis was performed to show the intra-TMT and inter-TMT quantification reproducibility (**Figure S1**).

### PRM Validation

To evaluate the level of PLGLB1 in new samples, PRM analysis was performed on a TripleTOF 5600 mass spectrometer (AB Sciex, USA) equipped with an Ultra Plus NanoLC 2D HPLC system (Eksigent Technologies, USA). The PLGLB1 peptide EQQCVIMAENR was selected for the PRM assay. The synthesized stable isotope-labeled peptide with C13- and N15-labeled C-terminal R and carbamidomethyl modification of C was spiked into each trypsin-digested sample as an internal standard. The peptide mixture was transferred onto a trap column and then separated onto a 15 cm analytical C18 column (150 μm inner-diameter, 1.9 μm resin, ReproSil-Pur C18-AQ, Dr Maisch GmbH) *via* a 30 min non-linear gradient from 8% to 90% solvent B (0.1% FA in acetonitrile) at 600 nl/min.

The PRM raw files were analyzed by Skyline (version 4.1.0.18163) for peak integration and quantification of the targeted peptides. All peaks were inspected manually to ensure the correct detection of precursor and fragment ions. The intensities of endogenous peptides in each sample were normalized to a corresponding stable isotope-labeled internal standard for relative quantification.

### Statistical Analysis

The hierarchical cluster analysis (53) was performed using the R package “pheatmap” with Euclidean distance and the clustering method “ward.D.” The cluster result and heat map were displayed using the R packages “ape” and “pheatmap,” respectively. For differential analysis, Wilcox nonparametric test was performed, and proteins with |log2(FC)|>0.263 (fold change 1.2) and *P*- value less than 0.05 were taken as significantly changed. The ROC curve analysis of PLGLB1 was performed by SPSS (16.0), with the relative levels of PLGLB1 in serum samples quantified by the TMT experiment.

### Pathway Enrichment Analysis

The enrichment analysis was performed by the hypergeometric test based on the KEGG (Kyoto Encyclopedia of Genes and Genomes) annotation. To avoid the bias of blood features, the proteins identified in the serum samples in this study were used as the background. Significantly enriched pathways with a P-value less than 0.01 and no less than 2 protein numbers were further concerned. The protein interactions in **Figure 2** were analyzed on the String website (https://string-db.org/) and then displayed by Cytoscape.

### Cluster and Disease Progression Analysis

Proteins that were identified in all sample groups at the first time point were clustered using the R package mFuzz (54) into 16 group clusters. While proteins that were identified at all the three sampling time points were clustered into 9 time-point clusters (time-point 3 of S31 was excluded because this sample was collected in the most severe period of the disease course of the patient). Based on those clusters, proteins that changed with the severity of COVID-19, recovered at the convalescence stage, and significantly changed (t-test, FDR <0.05) between time-point 3/time-point 1 were used for enrichment analysis. KEGG pathway enrichment analysis was performed using WebGestalt (55) and the significance level was corrected with Bonferroni.

## Data Availability Statement

The mass spectrometry proteomics data in this study can be found in online repositories. The names of the repository/repositories and accession number(s) can be found below: iProX (https://www.iprox.org/) Project ID: IPX0002584001.

## Ethics Statement

The studies involving human participants were reviewed and approved by the Ethical Review Board of Chongqing General Hospital. Written informed consent from the participants’ legal guardian/next of kin was not required to participate in this study in accordance with the national legislation and the institutional requirements.

## Author Contributions

Conceptualization, WS, PL and CH. Sample collection and clinical data management, YX, JW, HY, and CH. TMT experiments, LS and BF. Proteome data analysis, SW and JZ. Analysis results interpretation, WS, XJ, and YL. PRM experiments, XR and JZ. Data uploading, XR. Writing—Original Draft, WS, with input from co-authors. Writing—Review & Editing, WS and XJ. All authors listed have made a substantial, direct, and intellectual contribution to the work and approved it for publication.

## Funding

This study was supported by the National Megaprojects for Key Infectious Diseases (2018ZX10732202-003).

## Conflict of Interest

Author LS was employed by company Beijing Qinglian Biotech Co., Ltd. Author LS is shareholder of Beijing Qinglian Biotech Co., Ltd.

The remaining authors declare that the research was conducted in the absence of any commercial or financial relationships that could be construed as a potential conflict of interest.

## Publisher’s Note

All claims expressed in this article are solely those of the authors and do not necessarily represent those of their affiliated organizations, or those of the publisher, the editors and the reviewers. Any product that may be evaluated in this article, or claim that may be made by its manufacturer, is not guaranteed or endorsed by the publisher.

## Supplementary Material

Supplementary Figure 1 Scatter plots and Spearman’s correlation coefficients (represented by R) for the two replicate proteomic data **(A)** within TMT (Intra-TMT) and **(B)** between TMT (Inter-TMT).
