# Supplementary figures and images for: Longitudinal Serum Proteome Characterization of COVID-19 Patients With Different Severities Revealed Potential Therapeutic Strategies
Source: Front Immunol. 2022 Jul 26;13:893943. doi: 10.3389/fimmu.2022.893943 (PMC9361788; doi:10.3389/fimmu.2022.893943)

**A**

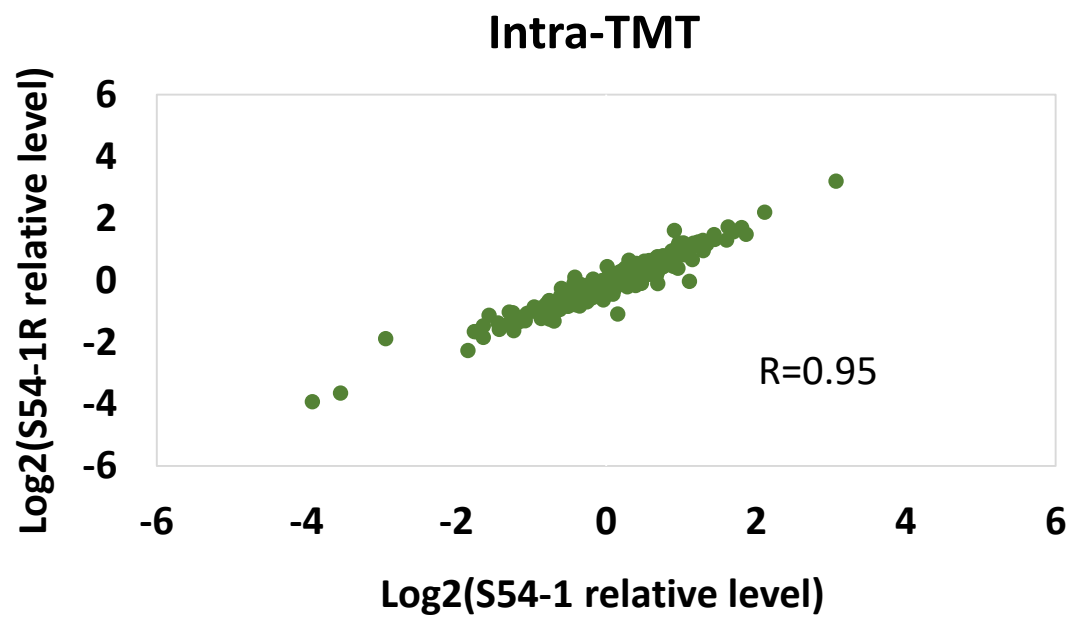

**B**

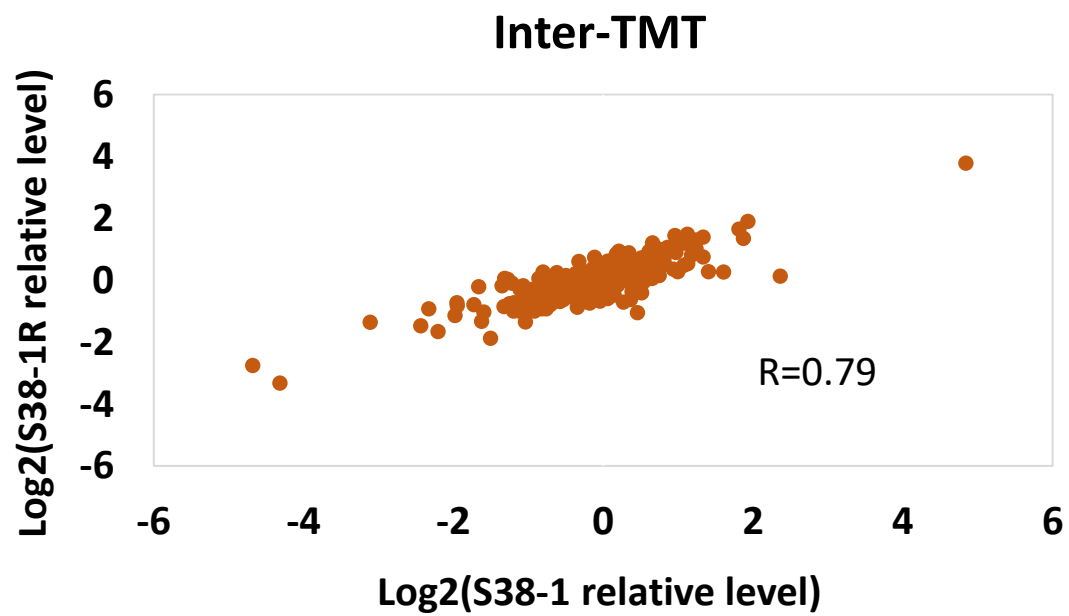

Supplement: Supplementary Figure 1 — Scatter plots and Spearman’s correlation coefficients (represented by R) for the two replicate proteomic data (A) within TMT (Intra-TMT) and (B) between TMT (Inter-TMT). [file Image_1.pdf]
