# Supplementary material for: Longitudinal Serum Proteome Characterization of COVID-19 Patients With Different Severities Revealed Potential Therapeutic Strategies
Source: Front Immunol. 2022 Jul 26;13:893943. doi: 10.3389/fimmu.2022.893943 (PMC9361788; doi:10.3389/fimmu.2022.893943)
Supplement: Supplementary file 5 [file Table_4.pdf]

**Table S3-1 Independent sample test of CD8+ T cell numbers (cells/μl) between severe and non-severe COVID-19 patients**

| | | Levene's Test for Equality of Variances | | t-test for Equality of Means | | | | | | |
|---|---|---|---|---|---|---|---|---|---|---|
| | | | | | | | | | 95% Confidence Interval of the Difference | |
| | | F | Sig. | t | df | Sig. (2-tailed) | Mean Difference | Std. Error Difference | Lower | Upper |
| CD8+ | Equal variances assumed | 2.601 | 0.112 | 2.785 | 58 | **0.007** | 166.12500 | 59.64348 | 46.73561 | 285.51439 |

**Table S3-2 Group Statistics**

| | Groups | N | Mean | Std. Deviation | Std. Error Mean |
|---|---|---|---|---|---|
| CD8+ | Non-severe patients | 48 | 4.0212E2 | 194.88858 | 28.12974 |
| | Severe patients (severe & critical) | 12 | 2.3600E2 | 133.34712 | 38.49400 |
